# Road-MobileSeg: Lightweight and Accurate Road Extraction Model from Remote Sensing Images for Mobile Devices

**DOI:** 10.3390/s24020531

**Published:** 2024-01-15

**Authors:** Guangjun Qu, Yue Wu, Zhihong Lv, Dequan Zhao, Yingpeng Lu, Kefa Zhou, Jiakui Tang, Qing Zhang, Aijun Zhang

**Affiliations:** 1 School of Mechanical and Electrical Engineering, Beijing University of Chemical Technology, Beijing 100020, China; 2022210351@mail.buct.edu.cn (G.Q.); 2023210455@buct.edu.cn (Y.L.); 2 Technology and Engineering Center for Space Utilization, Chinese Academy of Sciences, Beijing 100045, China; w8807246@163.com (Y.W.); zhoukf@ms.xjb.ac.cn (K.Z.); 3 College of Ocean Technology and Surveying, Jiangsu Ocean University, Lianyungang 222000, China; 2022220228@jou.edu.cn; 4 School of Information Science and Engineering, Shandong Agricultural University, Tai’an 271000, China; 2022110551@sdau.edu.cn; 5 College of Resources and Environment, University of Chinese Academy of Sciences, Beijing 100049, China; 6 Yanshan Earth Key Zone and Surface Flux Observation and Research Station, University of Chinese Academy of Sciences, Beijing 101408, China; 7 Institute of Aerospace Information Innovation, Chinese Academy of Sciences, Beijing 100045, China

**Keywords:** road extraction, deep learning, remote sensing, coordinate attention, mobile device

## Abstract

Current road extraction models from remote sensing images based on deep learning are computationally demanding and memory-intensive because of their high model complexity, making them impractical for mobile devices. This study aimed to develop a lightweight and accurate road extraction model, called Road-MobileSeg, to address the problem of automatically extracting roads from remote sensing images on mobile devices. The Road-MobileFormer was designed as the backbone structure of Road-MobileSeg. In the Road-MobileFormer, the Coordinate Attention Module was incorporated to encode both channel relationships and long-range dependencies with precise position information for the purpose of enhancing the accuracy of road extraction. Additionally, the Micro Token Pyramid Module was introduced to decrease the number of parameters and computations required by the model, rendering it more lightweight. Moreover, three model structures, namely Road-MobileSeg-Tiny, Road-MobileSeg-Small, and Road-MobileSeg-Base, which share a common foundational structure but differ in the quantity of parameters and computations, were developed. These models varied in complexity and were available for use on mobile devices with different memory capacities and computing power. The experimental results demonstrate that the proposed models outperform the compared typical models in terms of accuracy, lightweight structure, and latency and achieve high accuracy and low latency on mobile devices. This indicates that the models that integrate with the Coordinate Attention Module and the Micro Token Pyramid Module surpass the limitations of current research and are suitable for road extraction from remote sensing images on mobile devices.

## 1. Introduction

With the advancement in remote sensing technology and its widespread use, the extraction of roads from high-resolution remote sensing images has become increasingly crucial in various fields, such as automated navigation [1], autonomous driving [2], and urban planning [3]. While a higher resolution of remote sensing images has significantly improved the accuracy of extracted road information, it has also increased image data, thereby reducing the efficiency of image processing to some degree. Image processing efficiency will have a great impact on the efficiency of the extraction of roads. Additionally, there is an urgent requirement for portable and convenient road extraction equipment in practical engineering applications, especially in outdoor environments, to improve work efficiency. Therefore, the accurate and rapid extraction of road data from high-resolution remote sensing images and its implementation on portable devices (e.g., mobile devices) present a present-day challenge.

Road extraction from high-resolution remote sensing images involves two main aspects: (1) the extraction of road regions [4,5,6] and (2) the extraction of road centerlines [7,8,9,10]. The road centerline is typically extracted from the road region using algorithms, such as morphological refinement, in digital image processing technology [11]. This process is straightforward and can yield satisfactory results. Therefore, current research on road extraction focuses on extracting road regions from remote sensing images. Road region extraction methods generally fall into two categories [12]: traditional methods and deep learning-based methods.

Traditional methods typically involve methods based on digital image processing and classical machine learning. They manually define the type of road based on certain features and construct the corresponding feature extraction model to perform road extraction [13,14]. In the digital-image-processing-based methods, Valero et al. [15] introduced path opening and path closing according to mathematical morphology to extract structural pixel information from images, and employed advanced directional morphological operators to extract road information, which can be used to extract curved-shape roads. Garcia-Garcia et al. [16] used binary segmentation based on statistical evaluation of textures to extract roads, and could effectively identify regular roads in urban areas. Common methods for road extraction using classical machine learning algorithms include Support Vector Machine (SVM) [17], random forest (RF) [18], clustering [19], and decision tree models [20]. SVMs have demonstrated excellent high-dimensional data processing capabilities and strong robustness to successfully solve road extraction problems from high-resolution remote sensing images. Soni et al. [17] proposed a supervised multistage framework that uses a least-squares SVM (LS-SVM) to categorize image regions into two parts: road and non-road. The framework was designed to improve classification accuracy and minimize errors in extracting roads from images. Random forest models require relatively few adjustment parameters and generally only require setting parameters such as the number and depth of trees, which are easy to train. For example, Xu et al. [18] converted road networks from lines to polygons and used polygonal geometric descriptors to train a random forest classifier and identify candidates, then applied the model to extract roads. Clustering is usually used for unsupervised learning, where the dataset is not labeled, leading to significant time savings, e.g., Fengping et al. [19] examined an enhanced neighborhood FCM (fuzzy C-means) algorithm for extracting road regions. In this algorithm, the spatial distance and gray-level difference are calculated based on neighborhood characteristics, which are used as parameters of the objective function. The objective of this algorithm is to eliminate noise and improve the accuracy of road extraction. Zheng et al. [20] proposed a decision tree-based road recognition method. In this method, point cloud data, which are acquired by the roadside LiDAR sensors with low vertical resolution, are projected onto a plane rasterized to grids of points, and these grids are first classified into background grids and road grids using a decision tree. Finally, the accurate road boundaries are obtained by a minimum circumscribed rectangle algorithm. This method can perform road recognition more accurately and faster.

Traditional road extraction methods are valued for their low computational resource requirements, high stability, and fast speed. However, traditional methods become challenging when dealing with irregular road shapes and varying road surface colors and materials, making them unsuitable for large-scale road extraction [21,22].

In recent years, many studies have applied deep learning methods to extract roads from high-resolution remote sensing images due to their exceptional ability in image segmentation. This approach typically transforms the road extraction problem into a semantic segmentation problem in computer vision and automatically acquires road information [23,24]. Because of its robust generalization, this method is well-suited for large-scale implementation, which has contributed to its growing popularity in current research on road extraction from remote sensing images. In the context of deep learning-based road extraction, because model performance directly affects the quality of road extraction results, numerous studies have focused on improving model performance for various applications.

Some researchers have used convolutional neural networks to extract roads directly from raw remote sensing images [25,26]. Lu et al. [27] applied multiscale feature integration within a neural network to improve the robustness of road extraction. U-Net [28], an improved convolutional neural network model, has brought about better results in image segmentation. It can gradually reconstruct image resolution and capture intricate local details, image shapes, and global features while compressing the image. In addition, it can process global and local information simultaneously, making it effective in tasks such as medical image segmentation and road extraction. At present, more researchers have made further improvements in road extraction. Shao et al. [29] integrated spatial and channel attention mechanisms into the U-Net framework. The spatial attention mechanism was applied to extract road-related spatial details, whereas the channel attention mechanism was used to adjust the spectral characteristics of remote sensing images. Furthermore, Sultonov et al. [30] replaced the conventional convolutional layers in U-Net with ConvMixer layers, which significantly reduced the computations of the model. In addition, Alshaikhli et al. [31] combined U-Net and residual blocks and used fewer convolutional layers, achieving better prediction results than the standard U-Net model. Yu et al. [32] proposed EnRDeAU-Net, where the encoder is composed of input channels of residual U-Net Blocks, and the decoder is composed of attention gates in the output channels, which enables the effective extraction of roads from images with complex noise. Hou et al. [33] introduced the multiscale dense dilated convolution into U-Net, significantly enhancing its ability to detect hidden or obscured roads.

Nowadays, attention mechanisms have been incorporated into many deep learning-based models for road extraction to segment the road contour more accurately. Hu et al. [34] proposed a multiscale deformable Transformer network that can capture more comprehensive features than traditional Transformers in road extraction. Xie et al. [35] used the efficient LinkNet as the basic architecture and incorporated the Middle Block between the Encoder and Decoder to preserve both the global context semantic information and the information across different feature channels, resulting in a significant increase in extraction accuracy. In certain mountainous regions, road extraction has become even more challenging because of the interference of the road-like terrains and the shadows cast by mountains. Xu et al. [36] used an improved DSDNet to successfully extract roads in mountainous regions. Shao et al. [37] combined atrous convolutions with a pyramid pooling module to integrate multilevel features, making the information extraction more comprehensive. Wan et al. [38] proposed a dual-attention road extraction network, which could effectively solve the problem of incomplete and incoherent roads due to object occlusion. Li et al. [39] used the global attention module to acquire contextual information about the road, thereby improving the integrity of the road area.

Although deep learning technology is currently popular in road extraction, mainstream deep learning models typically have a large number of parameters, which leads to increased storage and computing resource consumption on devices. In particular, models with better performance often require more resources, which cramps their widespread applications [40,41]. This limitation is particularly evident in mobile devices that have limited memory, lower computational capabilities, and slower processing speed [42,43,44,45,46,47]. Some researchers are now working on improving the lightweight design of the model to decrease its size and computations while still maintaining its performance. Liu et al. [40] introduced depth-wise separable convolution to reduce the computations of the model and decrease the number of parameters. However, they only considered the parameters of the model, excluding the amount of calculation. Liu et al. [41] constructed a lightweight decoder using the transposed convolution and skip connections, reducing both the number of parameters and the amount of computation required by the model. Nevertheless, these studies only focus on reducing model parameters and computations without investigating portability to mobile devices. Currently, several lightweight segmentation models based on deep learning have been proposed for adaptation to mobile devices [42,43,44,45,46,47]. However, it is unclear whether these models can effectively extract road information on mobile devices, as there are no research results demonstrating their application to road extraction to date.

In summary, deep learning has achieved remarkable results in extracting roads from remote sensing images in some studies. However, the following issues still persist:

(1) While deeper and more complex deep learning networks often achieve better model performance [48,49], they also tend to consume more resources during loading and running procedures and typically require a large amount of computations that exceed the computing capacity of embedded devices, negatively impacting operational efficiency. These characteristics have hindered the implementation of current models for extracting roads on mobile devices with limited computing capabilities.
(2) The lightweight design of deep learning models is a practical and efficient approach to enable their implementation on mobile devices and ensure smooth operation with limited computational resources. However, it is uncertain whether the existing lightweight road extraction models [40,41] are suitable for deployment on mobile devices because they were not specifically developed for mobile applications, even though they can reduce model parameters and calculations to some extent. Meanwhile, the effectiveness of current lightweight segmentation models for mobile applications in extracting road information on mobile devices has not been further verified [42,43,44,45,46,47].

In short, none of the current lightweight models have been validated to effectively perform road extraction when deployed on mobile devices. This highlights the urgent need to develop a mobile-friendly lightweight model for extracting roads from remote sensing images without sacrificing extraction accuracy [46,47].

In this study, to address the limitations of current research, we propose a mobile-friendly, accurate, and lightweight model, Road-MobileSeg, which consists of two main components: Road-MobileFormer and Segmentation Head. This model is characterized by fewer parameters, less computation, and high accuracy, making it suitable for road extraction from remote sensing images on mobile devices. The main contributions and advantages of this study are summarized as follows:

1. A model for extracting roads, called Road-MobileSeg, has been developed. It is designed to be used on mobile devices and can extract roads from remote sensing images end-to-end.
2. The Road-MobileFormer, which serves as the backbone structure of Road-MobileSeg, was developed. It consists of an improved Token Pyramid Module and several Coordinate Attention Modules, which can achieve lightweight model structure and high accuracy of road extraction, thus ensuring smooth operation on mobile devices.
3. Three model structures, named Road-MobileSeg-Base, Road-MobileSeg-Small, and Road-MobileSeg-Tiny, were designed with different levels of complexity according to their backbone structures to adapt to the needs of mobile devices with different memory capacity and computing power.
4. Latency tests for different models on mobile devices with a CPU processor were conducted to validate the effectiveness and feasibility of our suggested models.

The rest of this paper is organized as follows. Section 2 presents the details of the proposed method. Section 3 gives the experimental configurations and experimental results. Finally, comprehensive discussions are presented in Section 4 followed by our concluding remarks in Section 5.

## 2. Methodology

### 2.1. Overall Architecture

**Figure 1:**
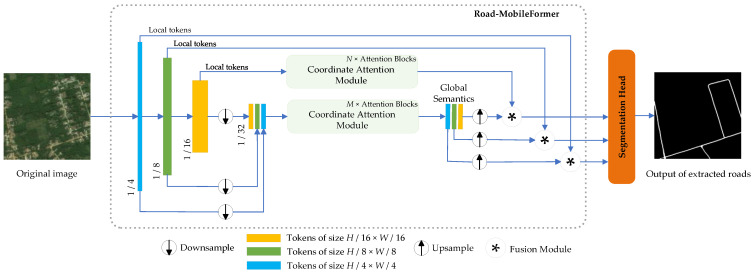
Architecture of Road-MobileSeg. *M* and *N* are the number of Coordinate Attention Blocks. The blue arrow indicates the execution of the next step.

Figure 1 depicts the overall structure of Road-MobileSeg, which consists of a Road-MobileFormer and a Segmentation Head. The Road-MobileFormer, which is used as the backbone structure of Road-MobileSeg, comprises three modules: The Micro Token Pyramid Module, the Coordinate Attention Module, and the Fusion Module. The Micro Token Pyramid Module is used to extract local features from an image, thus generating local feature maps, i.e., local tokens. In road extraction, incorporating the position information of roads will greatly improve the accuracy of the extraction result; therefore, the Coordinate Attention Module in Road-MobileSeg enables the model to fully capture and use the position information of roads. The Coordinate Attention Module, which is composed of multiple Coordinate Attention Blocks, is used twice in Road-MobileSeg. The Fusion Module then integrates both local features and global semantics, enabling the tokens to effectively incorporate rich spatial and semantic information. The Segmentation Head consists of two convolutional layers and uses the fused information as the input to produce the final segmentation image.

### 2.2. Micro Token Pyramid Module

**Figure 2:**
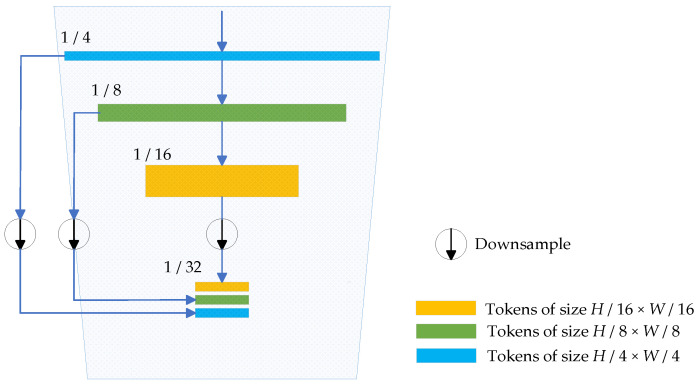
Architecture of the Micro Token Pyramid Module. The blue arrow indicates the execution of the next step.

We adopt the Micro Token Pyramid structure, which is lighter than the Token Pyramid in TopFormer [42] because it uses fewer local tokens, to extract local features from the image and generate corresponding local feature maps, as depicted in Figure 2. The Micro Token Pyramid Module uses the input image to create local tokens of different sizes. These tokens are then downsampled to new tokens of uniform size and connected along the channel dimension to generate scale-aware global semantics, which we refer to as scale tokens. In this paper, the average pooling technique is used to perform downsampling to generate uniformly sized new tokens. These newly connected tokens are used as input to the Coordinate Attention Module.

Specifically, the Micro Token Pyramid structure consists of stacked MobileNetV2 Blocks (as described in [50]), and takes an image, *I* ∈ *R^C×H×W^*, as input. Here, *C* represents the RGB channels with *C* = 3, *H* represents the image height, and *W* represents the image width. The image passes through these MobileNetV2 Blocks to generate local tokens of size *H*/4 × *W*/4, *H*/8 × *W*/8, and *H*/16 × *W*/16, respectively. These local tokens are then average pooled into new tokens with the target size, i.e., *H*/32 × *W*/32. Although using fewer tokens may neglect certain local feature information and potentially result in a slight decrease in processing accuracy, it can effectively reduce the number of parameters and computations. Because tokens from the Micro Token Pyramid Module are used as input for the Coordinate Attention Module, the computation required by the Coordinate Attention Module will consequently decrease, leading to a substantial reduction in the overall computational requirements of the model.

### 2.3. Coordinate Attention Module

In Road-MobileSeg, coordinate attention is used to improve model performance because the position information of roads is crucial for road extraction. Coordinate attention [51] not only focuses on channel information but also embeds position information into the attention module, enhancing the model’s attention. The Coordinate Attention Module is composed of stacked Coordinate Attention Blocks, which can be viewed as computational units to enhance the expressive power of the learned features for the model. In a certain range, increasing the number of attention blocks would result in more attention computations for the tokens at the current scale, thereby enhancing the model’s ability to capture position information and improving its accuracy. However, this would also increase the model’s complexity.

Applying the Coordinate Attention Module to local tokens of every scale can provide more abundant location information, which may improve the accuracy of road extraction to some degree. However, this would greatly increase the model’s complexity, resulting in excessive computational burden and impacting its usability on mobile devices. Hence, it is exclusively employed on the local tokens of size *H*/16 × *W*/16 in this study. Therefore, the Coordinate Attention Module is employed twice in Road-MobileSeg: once for the scale tokens to obtain scale-aware global semantics, and once for the local tokens to capture the position information of roads and to yield position-related tokens, which are taken as input for the Fusion Module like other local tokens. Although only two Coordinate Attention Modules are deployed in the model, the accuracy of the model was verified through multiple experiments, indicating that the setting of the attention modules in this study is reasonable and feasible.

**Figure 3:**
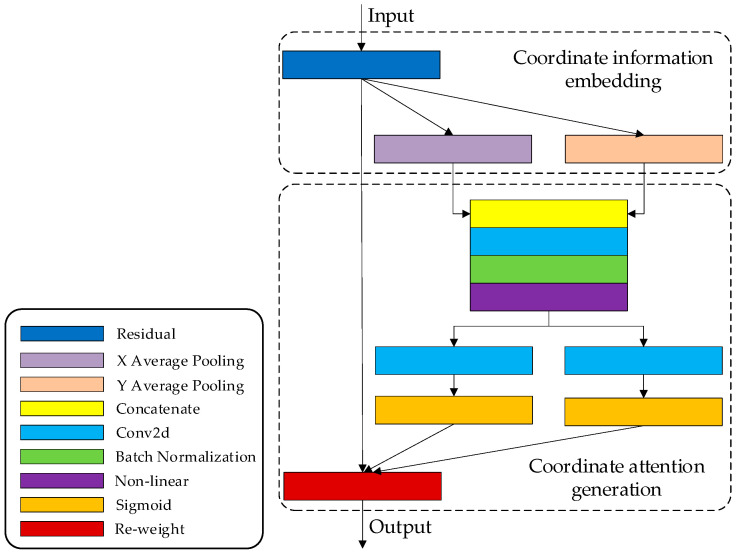
Architecture of the Coordinate Attention Block.

Coordinate attention encodes both channel relationships and long-range dependencies with precise position information in two steps: coordinate information embedding and coordinate attention generation. The structure of the Coordinate Attention Block is shown in Figure 3.

#### 2.3.1. Coordinate Information Embedding

To enhance the model’s ability to capture global information, we employ global pooling during coordinate information embedding. This process integrates all position information from the entire feature map into a global feature vector, while also significantly reducing the number of model parameters. This approach is commonly applied in channel attention to globally encode spatial information; however, it squeezes global spatial information into a channel descriptor, making it difficult to preserve positional details. Therefore, global pooling is decomposed into a pair of coordinate feature encoding operations to prompt attention blocks to capture long-range interactions spatially with precise position information.

For an input feature map *X*, we encode each channel by applying kernels of dimensions (*H*, 1) and (1, *W*) along the horizontal and vertical directions, respectively. The resulting output for the *c*-th channel at height *h* is formulated as

(1) $$z_{c}^{h}\left(h\right)=\frac{1}{W}\sum_{0\leq k<W}x_{c}\;(h,\;i)$$

where *x_c_* (*h*, *i*) represents the value of the feature map at height *h* and width coordinate *i* for channel *c*; *W* represents the width of the feature map.

Similarly, the output for the *c*-th channel at width *w* is expressed as

(2) $$z_{c}^{w}\left(w\right)=\frac{1}{H}\sum_{0\leq j<H}x_{c}\;(j,\;w)$$

where *x_c_* (*j*, *w*) represents the value of the feature map at height *j* and width coordinate *w* for channel *c*; *H* represents the height of the feature map.

The above two transformations correspond to X Average Pooling and Y Average Pooling in Figure 3, which aggregate features along the two spatial directions respectively, yielding a pair of direction-aware feature maps and facilitating the model’s capturing of position information.

#### 2.3.2. Coordinate Attention Generation

The two transformations, expressed as Equations (1) and (2), respectively, consolidate features along two spatial directions, creating a pair of direction-sensitive feature maps. The purpose of coordinate attention generation is to leverage the expressive representations that result from the transformations. The two feature maps are concatenated and subjected to a 1 × 1 convolutional transformation to yield *f*. After undergoing batch normalization (BatchNorm) and non-linear activation, *f* is partitioned into two separate tensors, *f^h^* and *f^w^*, along the spatial dimension. Subsequently, *f^h^* and *f^w^* are individually transformed into tensors, *g^h^* and *g^w^*, with the same number of channels as the input *X* by applying a 1 × 1 convolutional layer and an activation function for each. Afterward, *g^h^* and *g^w^* are expanded and used as attention weights, respectively. Finally, the output *Y* of the Coordinate Attention Block, which is of the same size as X, can be denoted as

(3) $$y_{c}\left(i,\;j\right)=x_{c}\;\left(i,\;j\right)\;\times {\;g}_{c}^{h}\;\left(i\right)\;\times \;g_{c}^{w}\;\left(j\right)$$

where *x_c_* (*i*, *j*) represents the value of the feature map at height *i* and width coordinate *j* for channel *c*; $g_{c}^{h}$(*i*) represents attention weights on the height dimension, and $g_{c}^{w}$(*j*) represents attention weights on the width dimension.

### 2.4. Fusion Module and Segmentation Head

After acquiring the scale-aware global semantics, we cannot directly add the local tokens obtained from the Micro Token Pyramid Module with the global semantics obtained from the Coordinate Attention Module due to the semantic gap between them. The Fusion Module, therefore, helps alleviate the semantic gap during the fusion process.

**Figure 4:**
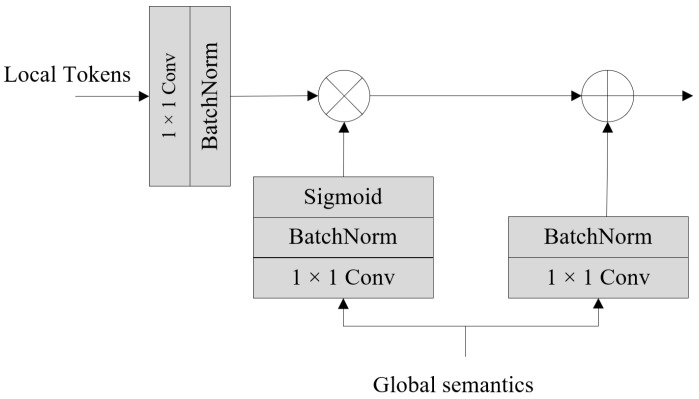
Architecture of the Fusion Module.

Figure 4 shows the structure of the Fusion Module, which takes the local tokens from the Token Pyramid Module and the global semantics from the Coordinate Attention Module as input. The local tokens undergo processing via a 1 × 1 convolution and a BatchNorm operation to create the feature to be injected. Global semantics sequentially pass through a 1 × 1 convolutional layer, a BatchNorm layer, and a sigmoid layer, generating semantics weights. Meanwhile, the global semantics also pass through a 1 × 1 convolutional layer, followed by a BatchNorm layer, to obtain the corresponding semantic feature. The three outputs above are of the same size. Subsequently, the global semantics are injected into the local tokens via Hadamard production and added to the feature obtained by Hadamard production, ultimately achieving the fusion between local tokens and global semantics. The outputs of multiple Fusion Modules share an equal number of channels, denoted as *C*. After the fusion process, the augmented tokens from diverse scales capture both spatial and semantic extensive information, which is critical for semantic segmentation. Additionally, the semantic gap among tokens is alleviated greatly in the fusion process.

**Figure 5:**
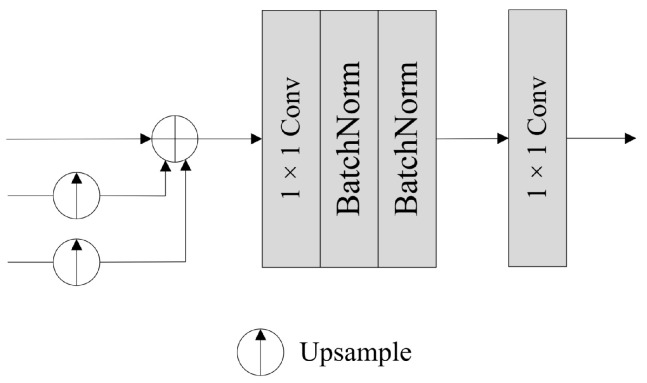
Architecture of the Segmentation Head.

In the segmentation stage, we apply the Segmentation Head structure from TopFormer [42] (see Figure 5). First, we take the three sets of tokens acquired from global semantic fusion as input, and upsample the low-resolution tokens with sizes of *H*/8 × *W*/8 and *H*/16 × *W*/16 to the same size as the high-resolution tokens, i.e., *H*/4 × *W*/4, as illustrated in Figure 1. Then, we combine the three groups of tokens from all scales by element-wise addition. Finally, the feature passes through a 1 × 1 convolutional layer, two BatchNorm layers, and another 1 × 1 convolutional layer to restore the image to its original size, producing the final segmentation map.

### 2.5. Architecture Variants

To adapt Road-MobileSeg to the conditions of different mobile devices, we have designed three different model structures based on Road-MobileSeg by setting different numbers of the Coordinate Attention Blocks (i.e., *M* and *N*) and that of output channels of the Fusion Module (also known as the number of input channels in the Segmentation Head), namely Road-MobileSeg-Tiny, Road-MobileSeg-Small, and Road-MobileSeg-Base. The structures of the three models have different levels of complexity. Table 1 lists the detailed configurations of the three different model structures.

## 3. Experiments and Evaluation

### 3.1. Dataset

We validated the effectiveness of Road-MobileSeg using the Deep Globe Road Extraction dataset [52], where each image measures 1024 × 1024 pixels and the resolution of the image is approximately 0.5 m. The dataset contains 6226 labeled images in total, which were randomly divided into a training set of 4981 images and a testing set of 1245 images for model training in our experiments.

### 3.2. Experiment Settings

#### 3.2.1. Training Settings

During training, the model was trained using Adam with decoupled weight decay (AdamW) as the optimizer, where the weight decay was set as 0.01. The training was performed for 60 epochs under the following conditions: the batch size was set to 16, the polynomial decay learning rate scheduling strategy was adopted with an initial learning rate of 0.01, and the loss function adopted was CrossEntropyLoss. The GPU employed for model training was the Nvidia GeForce RTX 4090 with 24 GB of memory.

#### 3.2.2. Latency Test Settings

Latency tests were conducted on ARM-based devices equipped with a single Qualcomm Snapdragon 888 processor to gauge the implementation latency of the model on mobile devices.

### 3.3. Evaluation Metrics

The performance of Road-MobileSeg was compared with several state-of-the-art mobile terminal models, including TopFormer [42], Segformer [43], BiseNet [44], and PP-Liteseg [45], to evaluate its effectiveness in extracting roads from remote sensing images on mobile devices. Meanwhile, four evaluation metrics, namely Mean Intersection over Union (MIoU) [16], parameter count, Floating-Point Operations (FLOPs) [53], and latency, were selected. The first three metrics were used to evaluate the model’s performance, while the last one was used to measure and compare the model’s processing speed on different devices.

MIoU is a standard used to assess the accuracy of semantic segmentation by measuring the degree of overlap between the predicted and actual target regions of the entire dataset. It is a frequently used and vital evaluation metric. To calculate MIoU, we computed the Intersection over Union (IoU) for each class *k* in the original dataset and then calculated the average of these individual IoU scores. The IoU and MIoU are defined respectively as follows:

(4) $$\mathrm{IoU}\;=\frac{\mathrm{TP}}{\mathrm{TP}+\mathrm{FP}+\mathrm{FN}}$$

(5) $$\mathrm{MIoU}=\frac{1}{n}\sum_{k=1}^{n}{\mathrm{IoU}}_{k}$$

where TP is true positive, indicating that the model correctly detected the positive class targets, FP is false positive, indicating that the model incorrectly identified areas of the negative class as positive class targets, and FN is false negative, indicating that the model failed to detect the actual positive class targets. *n* represents the total number of categories.

The parameter count and the amount of computation are used to measure the overall complexity of a model, and the volume of these two metrics determines whether a road extraction model is suitable for mobile devices. An increase in parameter count will directly increase the model’s complexity. The amount of computation, measured in terms of FLOPs, reflects the demand for computing power and resources when running the model on devices. Latency is correlated with factors such as model structure, image preprocessing, model inference time, and device performance. It reflects the model’s running speed on the device, and lower latency indicates faster processing.

### 3.4. Visual Evaluation

According to the road network density, we classified the road networks with different images as sparse or dense, selecting two samples for visual evaluation, respectively.

**Figure 6:**
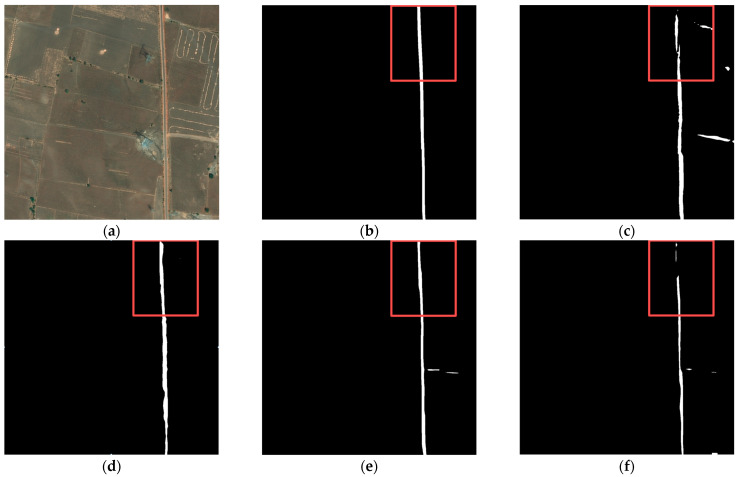

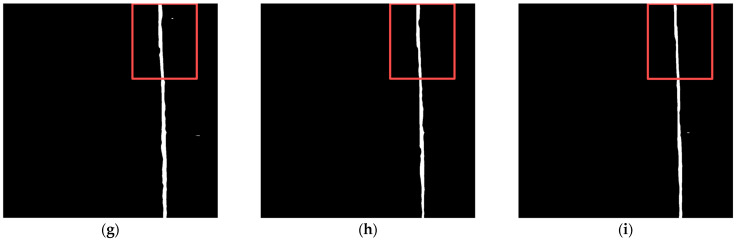
Road extraction results in the first set of sparse road networks. (**a**) original image; (**b**) ground truth; (**c**) Segformer; (**d**) TopFormer; (**e**) BiseNet; (**f**) PP-Liteseg; (**g**) Road-MobileSeg-Tiny; (**h**) Road-MobileSeg-Small; (**i**) Road-MobileSeg-Base. Areas for comparison with large differences are outlined in red boxes.

**Figure 7:**
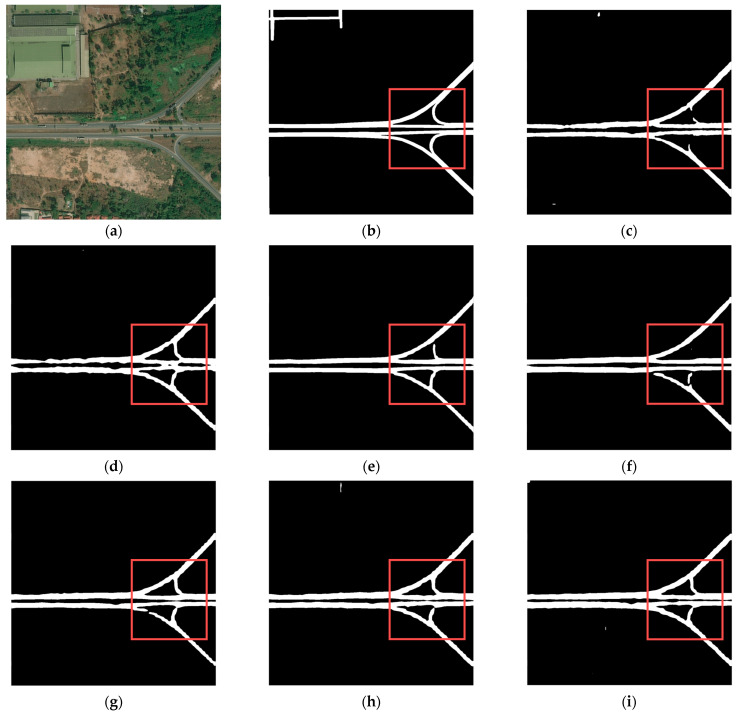
Road extraction results in the second set of sparse road networks. (**a**) original image; (**b**) ground truth; (**c**) Segformer; (**d**) TopFormer; (**e**) BiseNet; (**f**) PP-Liteseg; (**g**) Road-MobileSeg-Tiny; (**h**) Road-MobileSeg-Small; (**i**) Road-MobileSeg-Base. Areas for comparison with large differences are outlined in red boxes.

Figure 6 and Figure 7, show a sparse road network, respectively. In Figure 6, there is only one road, which is unobscured and clearly visible. All models could accurately extract the road, except for Segformer and PP-Liteseg. Both models are unable to extract a continuous road, resulting in incomplete road information. In Figure 7, compared with other models, the roads extracted by Road-MobileSeg-Base are the most complete and consistent with the ground truth. The roads extracted by other models exhibit different degrees of defects, particularly in the extraction results of TopFormer, where the two roads overlap in certain areas. Both sets of experiments demonstrate that the three models proposed in this paper acquired better results in extracting sparse road networks.

**Figure 8:**
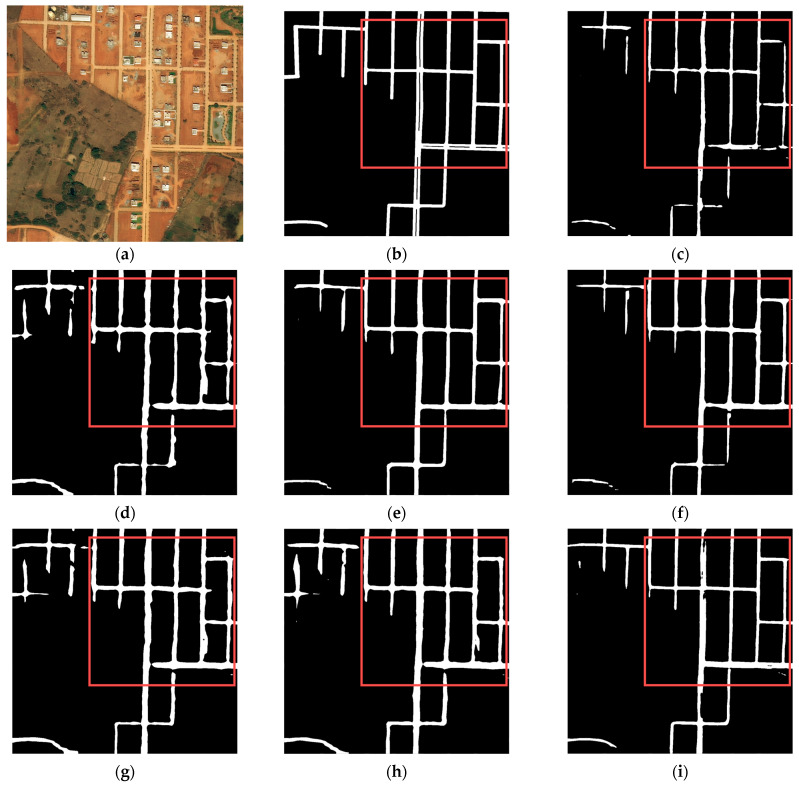
Road extraction results in the first set of dense road networks. (**a**) original image; (**b**) ground truth; (**c**) Segformer; (**d**) TopFormer; (**e**) BiseNet; (**f**) PP-Liteseg; (**g**) Road-MobileSeg-Tiny; (**h**) Road-MobileSeg-Small; (**i**) Road-MobileSeg-Base. Areas for comparison with large differences are outlined in red boxes.

**Figure 9:**
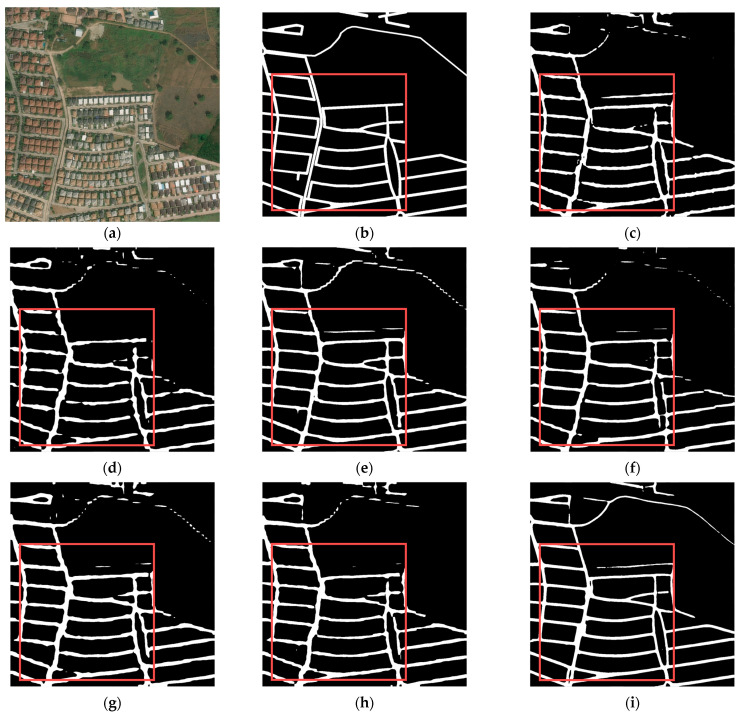
Road extraction results in the second set of dense road networks. (**a**) original image; (**b**) ground truth; (**c**) Segformer; (**d**) TopFormer; (**e**) BiseNet; (**f**) PP-Liteseg; (**g**) Road-MobileSeg-Tiny; (**h**) Road-MobileSeg-Small; (**i**) Road-MobileSeg-Base. Areas for comparison with large differences are outlined in red boxes.

Figure 8 and Figure 9 illustrate two different dense road networks. In Figure 8, the roads are regularly and neatly distributed. Discontinuity and width inconsistency of roads occur in the extraction results of TopFormer, while Segformer extracts roads with even more discontinuity. Compared with the two models mentioned above, the other models perform better. Figure 9 shows that as the road network complication increases (e.g., the number of roads increases and/or the shapes of roads become irregular), the accuracy of road extraction results for each model declines to varying degrees. However, BiseNet and Road-MobileSeg-Base still gain better extraction results compared with other models, and the extracted roads are the most consistent with the ground truth. The extraction effect is as good as BiseNet in that the coordinate attention is incorporated into Road-MobileSeg-Base, which makes the extracted road positions more accurate and exhibit better road continuity.

### 3.5. Quantitative Evaluation

Table 2 compares the quantitative results of different models. The MIoU of Road-MobileSeg-Base was the highest at 74.76%, followed by BiseNet with ResNet18 as the backbone, whose MIoU was 74.39%. The number of parameters determines the amount of memory occupied by the mobile device; therefore, fewer parameters result in less occupied memory. Road-MobileSeg-Tiny achieved the best result with a parameter count of only 1.41 M. On the other hand, BiseNet, which performed well in MIoU, had more than nine times as many parameters as Road-MobileSeg-Tiny. Road-MobileSeg-Tiny had the fewest FLOPs, followed by Road-MobileSeg-Small, Road-MobileSeg-Base, and TopFormer. BiseNet had a significant amount of computation with over 200 G FLOPs.

### 3.6. Latency Test on Mobile Devices

To test the effectiveness of our suggested model on mobile devices with a CPU processor, we employed PaddleLite to deploy the model on smartphones. PaddleLite is an open-source framework for deep learning, specifically engineered to streamline inferences on mobile, embedded, and IoT devices. It offers low-latency inferences for deep learning on the device.

The latency tests were conducted on a Xiaomi 11 smartphone equipped with a single Qualcomm 888 processor, running PaddleLite on a single thread. The Road-MobileSeg model was converted to a PaddleLite model using the PaddleLite opt tool during inference and then loaded onto the mobile device using PaddleLite in Android Studio. We used images with a resolution of 1024 × 1024 pixels from the DeepGlobe Road Extraction dataset as input for our experiments. When measuring the latency for each model, we averaged the values of 50 measurements and made it as the experimental result. Table 3 presents the latency test results for various models, allowing for easy comparison.

## 4. Discussion

In the visual evaluation results of Section 3.4, both BiseNet and Road-MobileSeg-Base performed well, as illustrated in Figure 6, Figure 7, Figure 8 and Figure 9. The primary factor contributing to BiseNet’s accurate road extraction is its utilization of a multi-path structure, i.e., the Spatial Path and the Context Path. The Spatial Path comprises three convolutional layers, capable of acquiring large spatial sizes of feature maps and preserving abundant spatial information. The Context Path employs a lightweight model like Xception and global average pooling to produce feature maps and provide sufficient receptive field, encoding high-level semantic context information. The Spatial Path generates low-level output features, while the Context Path generates high-level output features. These two types of features complement each other, which results in high accuracy of road extraction. The reason Road-MobileSeg can obtain accurate road extraction results is mainly due to its utilization of coordinate attention. Coordinate attention decomposes the global pooling operation into a pair of one-dimensional feature encoding operations along two spatial directions, known as encoding each channel separately along the horizontal and vertical coordinates, thus obtaining a pair of direction-sensitive feature maps. In this way, coordinate attention concentrates on channel information and position information concurrently, and embeds position information into the attention, enabling the capture of relationships between different positions in the input feature map and thereby refining the model’s focus. Furthermore, Road-MobileSeg employs two Coordinate Attention Modules. One module focuses on local tokens of size *H*/16 × *W*/16 to yield position-related tokens, while the other module focuses on scale tokens to acquire scale-aware global semantics. This manner further enhances the model’s ability to capture position information and allows attention blocks to capture long-range interactions spatially with precise position information.

PP-Liteseg simultaneously employs channel attention and spatial attention, allowing the model to capture both channel and spatial information. This improves the accuracy of semantic segmentation and enables the model to perform well in panoramic segmentation for urban environments. Unfortunately, although the amount of calculation is reduced and the speed of segmentation of the model is improved to some extent due to the reduction in the channels of high-level features and low-level features in the design of the Flexible and Lightweight Decoder, the accuracy of feature capturing decreases somewhat concomitantly, resulting in unsatisfactory road extraction results for wild areas compared with other methods, as shown in Figure 6f and Figure 7f.

While self-attention is incorporated into Segformer, the extracted roads using this model still suffer from inaccurate positioning because the model does not use positional encoding, as displayed in Figure 7c. In general, the self-attention mechanism facilitates the model to capture the relationships across various regions of the image and the positional relationships between spatial dimensions and channels. As a result, using the self-attention mechanism usually can improve the accuracy of road extraction to some extent. However, the Positional-Encoding-Free Design used in Segformer would cause the self-attention to fail in effectively capturing long-range dependencies of different regions in the image, leading to the loss of position information in the input sequence during road extraction. Therefore, when applying Segformer to road extraction that is sensitive to position information, the use of the Positional-Encoding-Free Design impairs the benefits of self-attention and leads to a drop in extraction accuracy.

TopFormer also utilizes a self-attention mechanism and incorporates an additional type of local token with a size of *H*/32 × *W*/32 compared with Road-MobileSeg. However, the road extraction accuracy of TopFormer is not superior to that of Road-MobileSeg-Base, as shown in Figure 8. This is because TopFormer only uses attention modules to generate global semantics, whereas Road-MobileSeg employs a Coordinate Attention Module for not only global semantics generation but also for local tokens of size *H*/16 × *W*/16. Despite using fewer local tokens than TopFormer, Road-MobileSeg achieves better overall performance by incorporating multiple Coordinate Attention Modules. This also indirectly demonstrates the superior effectiveness of the Coordinate Attention Module.

**Figure 10:**
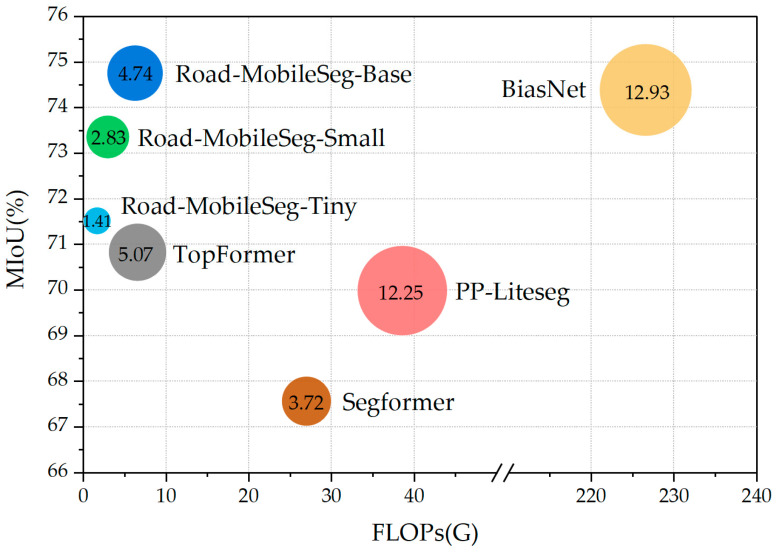
FLOPs and MIoU relationship.

Based on the quantitative evaluation results of the models in Section 3.5, a more intuitive comparison of the performance of each model can be obtained by examining the relationship between the MIoU and FLOPs of each model portrayed in Figure 10 using a bubble diagram. The bubble size represents the model parameter quantity, while the coordinates of the bubble center indicate the model’s FLOPs and MIoU values, respectively. As shown in Figure 10, all three model structures proposed in this study achieved high MIoU values, low parameter counts, and few FLOPs, demonstrating that the overall performance of these three structures surpasses that of other segmentation models when being applied to mobile platforms for road extraction from remote sensing images.

In Figure 10, both BiseNet and Road-MobileSeg-Base achieved high MioU, confirming the visual evaluation results in Section 3.4. Nevertheless, BiseNet has significantly more parameters and computations compared with Road-MobileSeg-Base. Specifically, the FLOPs of Road-MobileSeg-Base are only 1/36 of those of BiseNet. Therefore, Road-MobileSeg-Base outperforms BiseNet in overall performance. In BiseNet, the use of multiple paths, such as the Spatial Path and Context Path, and multiple convolutional layers in the Spatial Path can enhance the accuracy of road extraction. However, this also increases model complexity and computational burden. In particular, the configuration of convolutional layers in Spatial Path poses a dilemma. While employing more convolutional layers can undoubtedly enhance processing accuracy, it will also cause an increase in parameters and computational load. In contrast, both the Coordinate Attention Module and the Micro Token Pyramid Module in Road-MobileSeg have lighter structures, resulting in a significant reduction in model parameters and computations.

As for Segformer, the self-attention employed in it necessitates a significant amount of computation and causes Segformer to bear a sizable computational load on the whole, even though the Positional-Encoding-Free Design and the Lightweight All-MLP Decoder incorporated in Segformer can reduce the computational burden of the model to some degree. Conversely, Road-MobileSeg employs coordinate attention, which is less computationally intensive than self-attention from a computational load perspective.

Road-MobileSeg-Tiny achieved a similar MIoU value as TopFormer, but with 3.66 M fewer parameters and 4.89 G fewer FLOPs than TopFormer. This is attributed to the use of local tokens with respective size of *H*/4 × *W*/4, *H*/8 × *W*/8, *H*/16 × *W*/16, and *H*/32 × *W*/32 in TopFormer, and these local tokens are pooled into *H*/64 × *W*/64 in the Token Pyramid Module, while only local tokens of sizes *H*/4 × *W*/4, *H*/8 × *W*/8, and *H*/16 × *W*/16 are used in Road-MobileSeg and pooled into new tokens with the target size *H*/32 × *W*/32 in Micro Token Pyramid Module. As previously discussed, increasing the number of tokens used in TopFormer can contribute to improving accuracy to some degree in road extraction, but it also causes a significant increase in the model’s parameter quantity and computational load.

In PP-Liteseg, both channel and spatial attention are applied to enrich the fused feature representations. This is conducive to improving the accuracy of road extraction; however, it also results in an increase in the number of parameters and the computation of the model. Therefore, it is not as efficient as Road-MobileSeg in terms of making the model lightweight.

**Figure 11:**
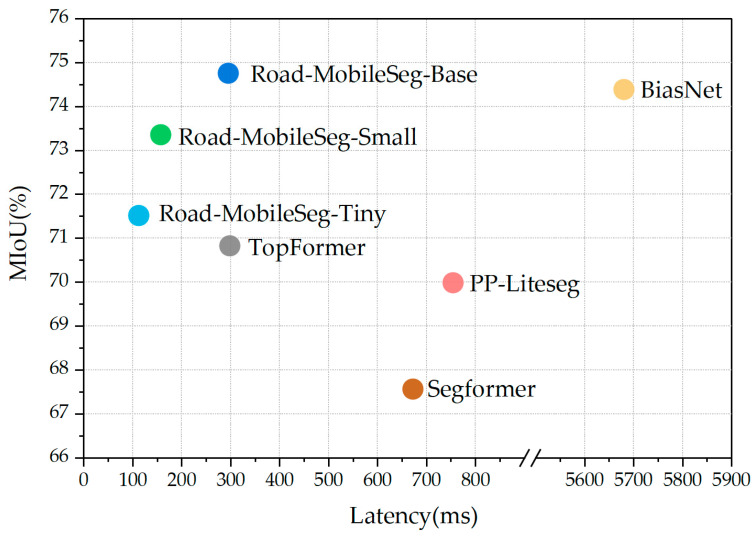
Latency and MIoU relationship.

Figure 11 shows a scatter diagram illustrating the relationship between latency and MIoU for each model on mobile devices, based on the results of the latency tests in Section 3.6. The diagram provides an intuitive demonstration of the performance of each model on mobile devices. The three model variants proposed in this study differ in the number of attention blocks and the output channels of the Fusion Module. This results in variations in parameter counts and computations for the three model structures, allowing them to adapt to different working scenarios. Specifically, the number of attention blocks configured in their respective attention modules is *M*/*N* = 2/2 in Road-MobileSeg-Tiny, *M*/*N* = 3/3 in Road-MobileSeg-Small, and *M*/*N* = 4/4 in Road-MobileSeg-Base. Compared with other models, these three model structures demonstrate superior overall performance with lower latency and higher MIoU. Consequently, they are more suitable for application on mobile devices. Among them, Road-MobileSeg-Base achieves the highest MIoU, whereas Road-MobileSeg-Tiny exhibits the lowest latency. When high accuracy is required on mobile devices, Road-MobileSeg-Base is the preferred option. Road-MobileSeg-Tiny is an ideal option when faster processing speed is necessary or when computational resources on mobile devices are limited. Road-MobileSeg-Small strikes a balance between accuracy and speed for road extraction on mobile devices, while requiring low computational resources; hence it is well-suited for situations requiring both speed and accuracy.

## 5. Conclusions

In this paper, we introduce a lightweight and accurate road extraction model from remote sensing images, namely Road-MobileSeg, that can be used to extract road information on mobile devices. Specifically, Road-MobileSeg consists of the following components: Micro Token Pyramid Module, Coordinate Attention Module, Fusion Module, and Segmentation Head. The Micro Token Pyramid Module greatly reduces the model’s complexity. The Coordinate Attention Module increases the accuracy of the extracted road location information. Three model structures were designed according to the number of attention blocks in the Coordinate Attention Modules and that of the output channels of the Fusion Module, i.e., Road-MobileSeg-Tiny, Road-MobileSeg-Small, and Road-MobileSeg-Base, with different model complexities to adapt to the limited memory and computational capacity of different mobile devices. The experimental results demonstrate that, compared with other models, our models have the advantages of lower computational load, fewer parameters, higher accuracy, and faster inference speed on mobile devices, making them suitable for mobile devices to complete road extraction work from remote sensing images.

In short, our research introduces an innovative approach for road extraction from remote sensing images, yielding a road extraction model with better overall performance that is suitable for mobile devices. However, in wilderness areas, there may be missed detection in remote sensing images due to vegetation occlusion and other reasons. This will most likely result in discontinuities in the roads extracted using our method, ultimately affecting road extraction results negatively. Future research should utilize the fusion of hyperspectral images, high-resolution remote sensing images, and other relevant data, such as digital elevation model (DEM) data, to gather additional information and reduce missed detections, thereby improving road extraction accuracy. The multichannel information of hyperspectral images will contribute to derive roads information in the regions obscured by vegetation, whereas DEMs can be used to address issues such as discontinuities in the extracted roads caused by local topography, by providing more detailed terrain spatial data through quantitative analysis. Furthermore, while the models proposed in this study demonstrated strong performance on the dataset used in this paper, it is recommended that additional datasets be employed in future work to evaluate the models’ efficacy not only in road extraction, but also in the extraction of other objects, such as panoramic segmentation in wild environments. Moreover, the architecture of the model should be refined to expand its attention mechanisms to enhance model accuracy and decrease the number of parameters and computations required, making it available for a wider range of remote sensing applications on mobile devices.

## Figures and Tables

**Table 1:** Configuration details of model structures.

Stage	Output Size	Tiny	Small	Base
Micro Token Pyramid Module	512 × 512	Conv, 3 × 3, 16, 2 MB, 3, 1, 16, 1	Conv, 3 × 3, 16, 2 MB, 3, 1, 16, 1	Conv, 3 × 3, 16, 2 MB, 3, 1, 16, 1
	256 × 256	MB, 3, 4, 16, 2	MB, 3, 4, 24, 2	MB, 3, 4, 32, 2
		MB, 3, 3, 16, 1	MB, 3, 3, 24, 1	MB, 3, 3, 32, 1
	128 × 128	MB, 5, 3, 32, 2	MB, 5, 3, 48, 2	MB, 3, 4, 64, 2
		MB, 5, 3, 32, 1	MB, 5, 3, 48, 1	MB, 3, 3, 64, 1
	64 × 64	MB, 3, 3, 64, 2	MB, 3, 3, 96, 2	MB, 3, 4, 128, 2
		MB, 3, 3, 32, 1	MB, 3, 3, 96, 1	MB, 3, 3, 128, 1
Coordinate Attention Module	32 × 32	*M*/*N* = 2/2	*M*/*N* = 3/3	*M*/*N* = 4/4
Fusion Module	256 × 256, 128 × 128, 64 × 64	*C* = 128	*C* = 192	*C* = 256

The size of the input is 1024 × 1024, (Conv, 3 × 3, 16, 2) indicates that Conv is a convolution layer with a 3 × 3 convolution kernel, its output channels = 48 and stride = 2. (MB, 3, 1, 16, 1) represents a MobileNetV2 Block with kernel size = 3, expand ratio = 1, output channels = 16 and stride = 1. *M*, *N* are the numbers of Coordinate Attention Blocks, and *C* is the number of output channels of the Fusion Module.

**Table 2:** Quantitative evaluation results.

Method	Backbone	Parameters (M)	FLOPs (G)	MIoU (%)
Segformer	MixVisionTransformer_B0	3.72	26.98	67.57
TopFormer	TopTransformer_Base	5.07	6.54	70.83
BiseNet	ResNet18	12.93	226.58	74.39
PP-Liteseg	STDC2	12.25	38.57	69.99
Road-MobileSeg-Tiny	Road-MobileFormer-Tiny	**1.41**	**1.65**	71.52
Road-MobileSeg-Small	Road-MobileFormer-Small	2.83	2.93	73.36
Road-MobileSeg-Base	Road-MobileFormer-Base	4.74	6.23	**74.76**

Note: Bold font indicates the best values of the respective columns.

**Table 3:** Latency test results of different models.

Method	Backbone	Latency (ms)
Segformer	MixVisionTransformer_B0	672
TopFormer	TopTransformer_Base	298
BiseNet	ResNet18	5680
PP-Liteseg	STDC2	754
Road-MobileSeg-Tiny	Road-MobileFormer-Tiny	**112**
Road-MobileSeg-Small	Road-MobileFormer-Small	157
Road-MobileSeg-Base	Road-MobileFormer-Base	295

Note: Bold font indicates the best values of the respective columns.

## Data Availability

The dataset used in this research is sourced from DeepGlobe, and it is openly accessible at https://www.kaggle.com/balraj98/deepglobe-road-extraction-dataset (accessed on 1 March 2023).
